# Data monitoring committees: history and their future

**DOI:** 10.1186/1745-6215-14-S1-I3

**Published:** 2013-11-29

**Authors:** David L DeMets

**Affiliations:** 1 Department of Biostatistics and Medical Informatics, University of Wisconsin, Madison, USA

## 

Data Monitoring Committees (DMCs), sometimes referred to as Data & Safety Monitoring Boards, have had a successful history over four decades in both government and industry sponsored trials. Their primary mission is to independently evaluate the accumulating data in an on-going clinical for the risks and benefits on behalf of trial participants, investigators, sponsors and regulators. DMCs can recommend that trials be terminated for overwhelming evidence of benefit, sufficient evidence of harm, futility or other external information. This process is quite challenging and requires a multidisciplinary team of experts who are independent from the trial investigators and sponsors.

Despite this long history of success, a number of new challenges for DMCs have emerged. These include a serious shortage of trained and experienced experts in clinical trial monitoring as more trials demand this oversight. There is a trend for data monitoring committee charters to become longer, more complex and appear similar to a legal document contract that must be strictly followed when experience indicates this is not often possible. DMCs are subject to liability risks often with no indemnification. Restrictions are being placed on what data can be reviewed and how often as well as what analyses methods must be used. More recently, DMCs are being asked to implement certain adaptive designs which may modify sample size targets based on emerging trends in a primary outcome despite being aware that the total data profile might not support such changes. These along with other challenges will be discussed.

